# Characterization of *Papaya ringspot virus* isolates infecting transgenic papaya ‘Huanong No.1’ in South China

**DOI:** 10.1038/s41598-018-26596-x

**Published:** 2018-05-29

**Authors:** Zilin Wu, Cuiping Mo, Shuguang Zhang, Huaping Li

**Affiliations:** State Key Laboratory of Conservation and Utilization of Subtropical Agro-bioresources, Guangdong Province Key Laboratory of Microbial Signals and Disease Control, College of Agriculture, South China Agricultural University, Guangzhou, 510642 China

## Abstract

In 2006, the release and cultivation of the genetically modified papaya cultivar ‘Huanong No.1’ successfully controlled the destructive papaya ringspot disease caused by *Papaya ringspot virus* (PRSV) in South China. However, some transgenic papaya plants from Guangdong and Hainan are found infected by PRSV. In this study, Field investigation was carried out and susceptible transgenic papaya samples were collected during 2012–2016. Twenty representative isolates were artificially inoculated into *Cucurbita pepo* and commercialised ‘Huanong No.1’ papaya, and results indicated that the plants showed obvious disease symptoms. Phylogenetic analysis of CP genes of 120 PRSV-infected isolates showed that PRSV can be divided into three groups. Isolates from Guangdong and Hainan belong to Group III, which is further divided into two subgroups. The isolates collected in this study have greatly diverged from the previously reported dominant strains Ys, Vb and Sm in South China, indicating that they belong to a new lineage. Further analysis showed a highly genetic differentiation between isolates, and 27.1% of the isolates were identified as recombinants on the basis of CP nucleotide sequences. These results indicate that the genetic variation of PRSV and the formation of the new virus lineage may explain the loss of transgenic papaya resistance in South China.

## Introduction

*Papaya ringspot virus* (PRSV) of the genus *Potyvirus* and the family *Potyviridae* is a threat to the papaya and cucurbit industries in tropical and subtropical regions. Papaya infected with PRSV exhibits symptoms such as leaf chlorosis and mosaic, water-soaked oily streaks on the petiole, ringspots on the fruit, young leaf distortion and even shoestring-like symptoms. Consequently, fruit production and quality can be severely decreased, and fruit sugar levels reduce by 50% or more. PRSV is grouped into papaya-infecting type-P (PRSV-P) and non-papaya-infecting type-W (PRSV-W). PRSV-P isolates infect plants of the families *Caricaceae*, *Cucurbitaceae* and *Chenopodiaceae*, whereas PRSV-W isolates infect plants of the families *Cucurbitaceae* and *Chenopodiaceae*^1^. PRSV-P and PRSV-W are serologically indistinguishable, but can be distinguished by specific hosts and are transmitted non-persistently by several species of aphids^2^. The virus has a positive single-stranded RNA of approximately 10 kb and encodes a polyprotein that is cleaved into 10 mature functional proteins^3^, namely, protein 1 protease, helper component protease, P3 protein, six kilodalton peptide, cytoplasmic inclusion, 6K2, viral protein genome-linked, nuclear inclusion A protease, nuclear inclusion b RNA-dependent RNA polymerase (NIb), coat protein (CP)^4^ and additional peptide P3N-PIPO^5^. Several reports on PRSV diversity, strain identification, phylogeny and recombination focused on the CP gene^6–10^.

From a global perspective, the most effective approach to prevent and control PRSV is to cultivate virus-resistant transgenic papaya cultivars. The world’s first commercially genetically modified papaya containing the PRSV CP gene was developed in Hawaii (USA) in 1998. Commercialised cultivars ‘SunUp’ and ‘Rainbow’ show high resistance to Hawaii PRSV isolates^11–13^. These two cultivars have been planted in Hawaii and remained resistant over the past two decades (personal communication). However, those transgenic papayas exhibit varying defence mechanisms against PRSV isolates from other geographical regions. For example, symptoms in papaya infected with isolates from the Bahamas, Florida or Mexico are delayed and mitigated, whereas those in papaya infected with isolates from Brazil, Thailand or Taiwan are also delayed but the resistance is eventually lost^14–16^. This finding implies that transgenic papaya only has defence mechanisms against local isolates.

Papaya is mainly planted in Hainan, Guangdong, Guangxi and Yunnan in South China. PRSV has become increasingly destructive and has seriously restricted the development of the papaya industry since it was first reported in China in 1959^17^. Cai *et al*.^18^ identified four PRSV isolates, Ys, Vb, Sm and Lc, in South China, based on the different symptoms on *Cucurbita pepo*. They collected and analysed 313 papaya virus samples in 1994 and showed that 40.57% of diseased plants are infected with Ys, 10.22% with Vb, 4.47% with Lc, 0.64% with Sm, and 44.08% with Ys and Vb mixed. This result indicated that Ys was the dominant strain, followed by Vb, with less distribution of Sm and Lc. A transgenic papaya cultivar, designated as ‘Huanong No.1’, carrying the *NIb* gene of PRSV Ys, was successfully generated from our laboratory in 1998^17^. After we got transgenic papaya, we collected different isolates from whole region of South China, including Guangdong, Hainan, Guangxi, Fujian provinces, where the transgenic papaya would be commercialized, and conducted resistance evaluation in greenhouse. The results showed that ‘Huanong No.1’ were highly resistant against all of these isolates. Then, we analyzed the resistant mechanisms and found that the resistance is due to the action of gene silencing^17,19–22^. Since the promotion of commercialisation in 2006, ‘Huanong No.1’ has shown a wide range of resistance to PRSV isolates in South China, and its annual planting area accounts for 85% of the total area of papaya^17^. However, we firstly observed that some plants of ‘Huanong No.1’ exhibited symptoms similar to those caused by PRSV in some plantations in Dongfang, Hainan in 2012. Since then, PRSV-susceptible transgenic plants have been found in other transgenic papaya plantations in Guangdong and Hainan, where a trend of devastating yield reduction for the papaya industry has been recorded. A few scholars have reported some PRSV isolates in Hainan^23,24^ but not in Guangdong, where ‘Huanong No.1’ is widely cultivated. In addition, these isolates have yet to be compared with the four dominant strains identified by Cai *et al*. in South China^18^. The variation of PRSV isolates or strains needs to be clarified after a long-term and extensive cultivation of the transgenic papaya. Aside from their different geographical environments, Guangdong and Hainan also support different papaya cultivars. Therefore, the PRSV isolates of the two regions should be systematically compared.

This study investigated PRSV isolates from Guangdong and Hainan during 2012–2016 to explain the loss of transgenic papaya resistance from the perspective of viruses and compared the genetic diversity and population genetic structure of these isolates to clarify the mechanism underlying virus evolution and population genetics. This study may serve as a theoretical basis for further prevention and control of PRSV.

## Results

### Field investigation of the virus infection

**Figure 1:**
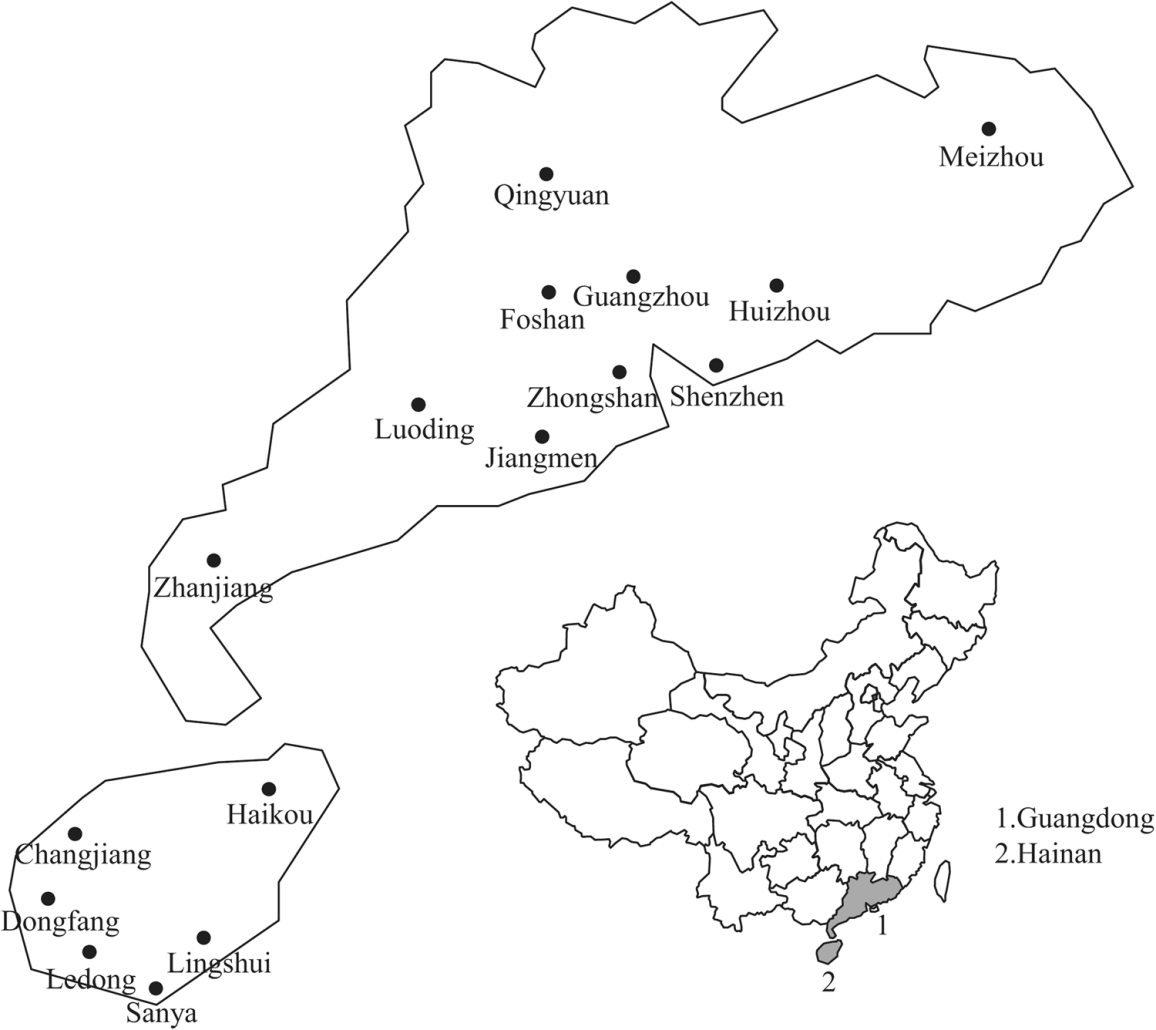
Skeleton drawing map showing the localities of the field investigation and sample collection of *Papaya ringspot virus* isolates in this study.

A total of 495 susceptible samples showing symptoms of leaf mosaic and malformation, streaks on stems and petioles, and ringspot on the fruits were collected from ‘Huanong No.1’papaya plantations in Guangdong and Hainan during 2012–2016 (Fig. 1). In 2016, the disease incidence in Guangdong was generally 5–30% but ≥60% in few seriously diseased areas, whereas that in Hainan was usually 80% but 90–100% in some areas. This finding suggests that ‘Huanong No.1’ papaya plants in South China are susceptible to viruses and that the disease incidence in Hainan is generally higher than that in Guangdong. Then, all of these samples were identified by RT-PCR and then by sequence analyses. Results showed that 128 of those samples were infected with PRSV, including 69 samples in Guangdong (41 in Guangzhou, 3 in Jiangmen, 10 in Zhanjiang, 6 in Zhongshan, 4 in Foshan and 1 in the rest of the regions) and 59 samples in Hainan (40 in Sanya, 15 in Dongfang and 4 in Ledong).

### Symptoms of squash and papaya inoculated with representative isolates

**Figure 2:**
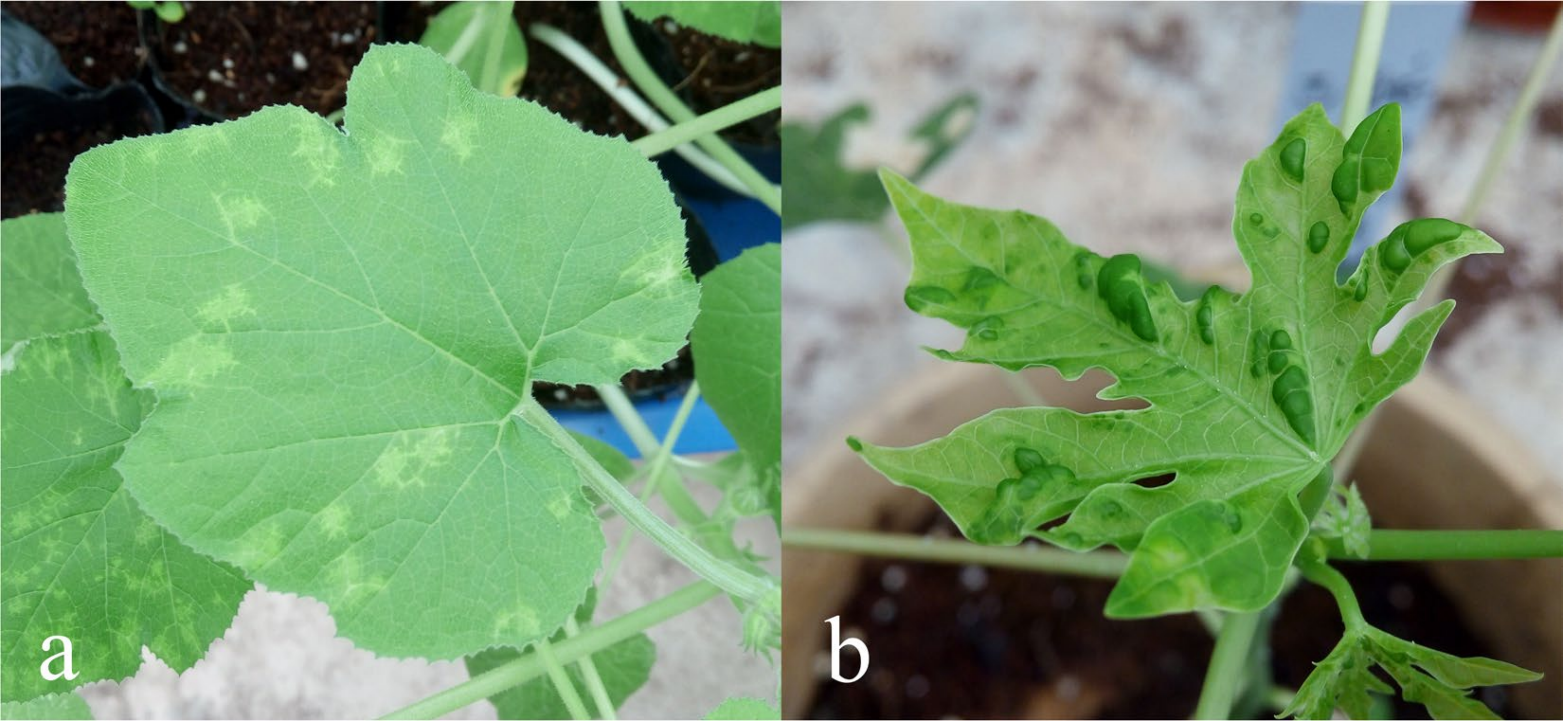
Different types of symptoms developed on *Cucurbita pepo* cultivar ‘Zaoqingyidai’ (**a**) and transgenic ‘Huanong No.1’ papaya (**b**) at 9 and 15 days after mechanical inoculation with one representative isolate of *Papaya ringspot virus*, respectively.

The 128 samples infected with PRSV isolates were divided into four groups according to geographical regions (Guangdong and Hainan) and leaf symptom characteristics (mosaic and distortion). Five samples were selected from each group, and 20 purified isolates were obtained by inoculation on *Chenopodium quinoa*. Then, isolates from the four groups were utilised to inoculate squash cultivar ‘Zaoqingyidai’ (*C*. *pepo*) and ‘Huanong No.1’ papaya. Results showed that all of the isolates caused similar viral symptoms in the inoculated plants of squash and papaya. In specific, irregular chlorosis along the leaf veins on squash, and distortion, irregular chlorosis and green islands on papaya were observed (Fig. 2). The RNAs of those diseased plants were extracted, and PRSV CP genes were cloned and sequenced. The amino acid sequences of the infected PRSV isolates were essentially coincident with those of the original inoculated isolates. These results reveal that the PRSV isolates obtained from South China can indeed infect transgenic papaya cultivar ‘Huanong No.1’ and break the resistance against PRSV in South China.

### Analysis of sequence variation in CP genes

Sequencing results showed that the CP genes of the 128 isolates in this study were approximately 864–867 bp in nucleotide length and were deposited in GenBank (Table 1S). Among the 128 isolates, 69 were from Guangdong and 59 from Hainan. In addition, five PRSV complete CP sequences originated from Hainan were retrieved from GenBank. Consequently, 133 complete CP sequences were included in the population genetic analysis of the virus. In the meantime, 23 complete CP sequences from different countries and regions, including the three previously reported dominant strains (Ys, Vb and Sm) in South China^18^, were downloaded from the GenBank as references (Table 1S).

**Figure 3:**
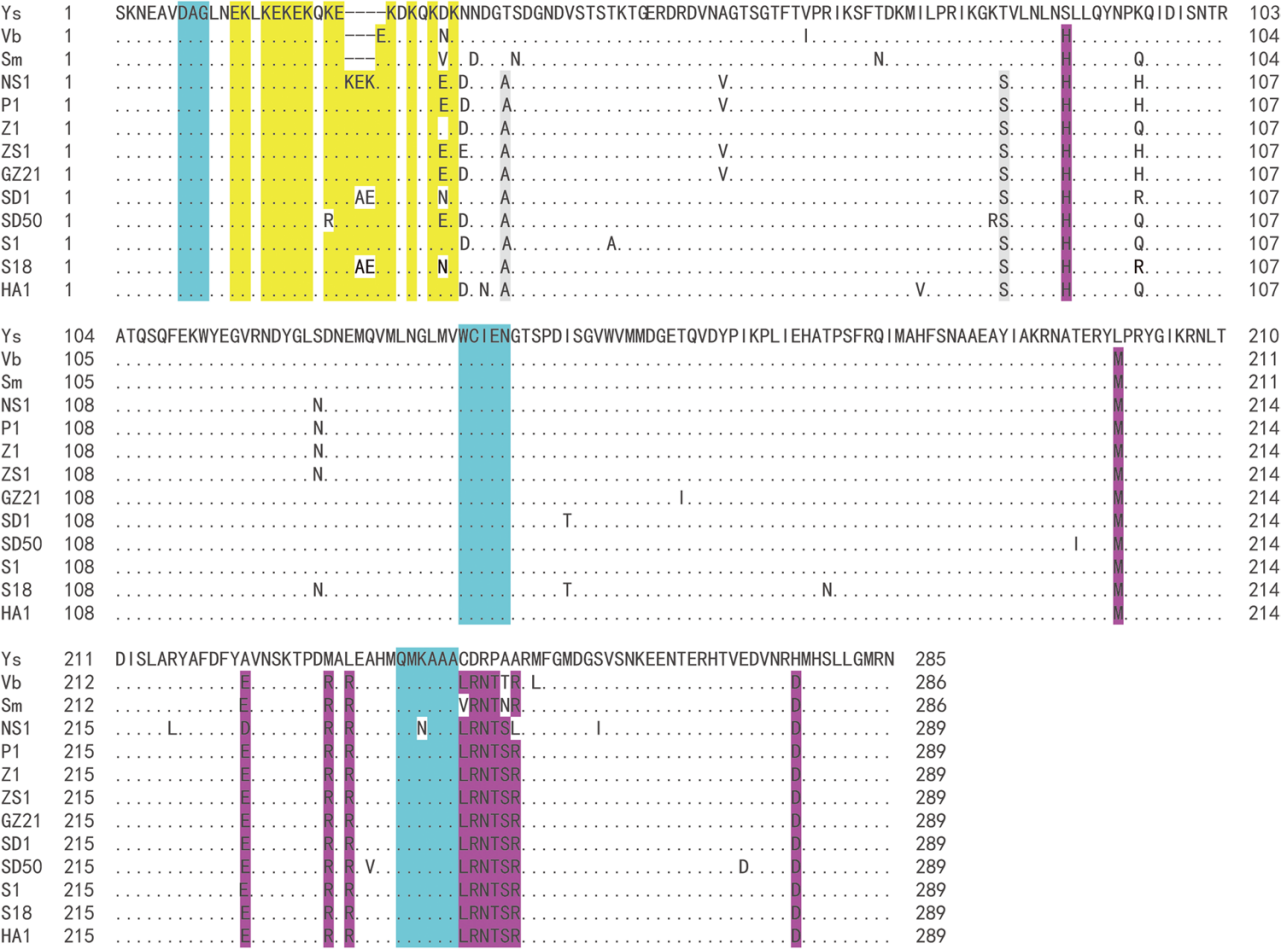
The amino acids sequences of Coat protein of *Papaya ringspot virus* isolates from South China showing the deletions (−) in the Ys, Vb, and Sm isolates, conserved DAG, WCIEN & QMKAAA domains (blue regions) and mutation sites. Ys, Vb, and Sm were the previously reported dominant strains in South China, NS1, P1, Z1, ZS1, and GZ21 are Guangdong isolates, while SD1, SD50, S1, S18, and HA1 are Hainan isolates. Different letters in the same column indicate different amino acids in that position. The yellow regions mean the glutamic acid and lysine (EK) repeat patterns, the gray and purple regions mean the different amino acids between this study’s isolates and three previously reported dominant strains in South China. Multiple alignments were performed with CLUSTALW program included in MEGA 6.

The identities of 69 PRSV isolates from Guangdong were 84.7–100% at the nucleotide level, whereas those of 64 isolates from Hainan were 92.3–100%. The identities of 133 isolates from South China were 84.2–93.9%, 86.5–97.6% and 86.3–97.7% compared with the three dominant strains (Ys, Vb and Sm), respectively. Furthermore, boxplot analysis showed that the identities between the three strains and all other isolates were 86.6–89.2%, 89.2–92.4% and 89.2–92.1%, respectively (Fig. 1S). These results indicated that the collected PRSV isolates differed from Ys but were relatively similar to Vb and Sm in South China. Therefore, five Guangdong isolates (NS1, P1, Z1, ZS1 and GZ21) and five Hainan isolates (SD1, SD50, S1, S18 and HA1), as well as Ys, Vb and Sm, were chosen for alignment analysis of the amino acid sequence of CP genes. A large number of EK (glutamic acid and lysine) repeats, including three conserved DAG, WCIEN and QMKAAA regions, were found in all isolates, but several domain sites of amino acid sequences showed a distinct variation (Fig. 3). Among them, three to four EK repeats were added and two sites were changed (Thr_34_ to Ala_38_ and Thr_82_ to Ser_86_) in the 10 Guangdong and Hainan isolates as compared with those in the three previously reported dominant strains (Ys, Vb and Sm). In addition, 12 sites of the amino acids showed variations (Ser_88_ to His_92_, Leu_200_ to Met_204_, Ala_223_ to Glu_227_, Met_231_ to Arg_235_, Leu_233_ to Arg_237_, Cys_244_ to Leu_248_, Asp_245_ to Arg_249_, Arg_246_ to Asn_250_, Pro_247_ to Thr_251_, Ala_248_ to Ser_252_, Ala_249_ to Arg_253_ and His_276_ to Asp_280_) in 10 isolates as compared with that in Ys, and two sites were different (Val_59_ to Ala_59_, Asn_127_ to Ser_127_) between the Guangdong and Hainan isolates.

Diversity analysis at the nucleotide level showed that 107 haplotypes were identified in the 133 complete sequences of CP genes with an overall haplotype diversity of 0.995 and a nucleotide diversity of 0.057 when the sequences from the Guangdong and Hainan isolates were considered together (Table 1). When the sequences were considered according to individual geographic location, higher nucleotide (0.054 vs. 0.045) but lower haplotype (0.986 vs. 0.995) diversity was found in Guangdong than in Hainan. These results indicated that the sequence differences of the Guangdong isolates were greater than those of the Hainan isolates, but the genetic diversity of population in Hainan was higher than that in Guangdong. Overall, the haplotype diversity in both regions was greater than 0.5 and the nucleotide diversity was higher than 0.005, indicating that the PRSV population in both regions had a high degree of genetic diversity.

**Table 1:** Sample sizes and genetic variation of *Papaya ringspot virus* populations from Guangdong and Hainan based on the CP gene nucleotide sequences.

Region	Sample size	Haplotypes	Haplotype diversity	Nucleotide diversity
GD	69	52	0.986	0.054
HN	64	55	0.995	0.045
All	133	107	0.995	0.057

GD, Guangdong isolates; HN, Hainan isolates; All, Guangdong and Hainan isolates. Haplotype diversity and nucleotide diversity were estimated using DnaSP 5.0.

### Analysis of genetic differentiation

Genetic differentiation of 133 CP gene sequences of the Guangdong and Hainan isolates showed that the *K*_*ST*_, *Snn* and *F*_*ST*_ of the CP gene sequences were 0.128, 0.985 and 0.227, respectively (Table 2). The P values of these indicator values from the outcome of 1000 Bernoulli trials were 0.000, which showed statistically significant differences at the 0.001 level. Hence, the null hypothesis that the two populations from Guangdong and Hainan are not genetically differentiated was rejected. The above three indicator values revealed a significant differentiation between the two PRSV populations from Guangdong and Hainan.

**Table 2:** Statistical tests for differentiation between two *Papaya ringspot virus* populations from Guangdong and Hainan based on the *CP* gene nucleotide sequences.

K_ST_	p	S_nn_	p	F_ST_	p
0.128	0.000***	0.985	0.000***	0.227	0.000***

ns, not significant; *0.01 < P < 0.05; **0.001 < P < 0.01; ***P < 0.001. *K*_*ST*_ and *S*_*nn*_ were implemented in DnaSP 5.10, while *F*_*ST*_ was in Arlequin 3.5. The hypothesis of deviation from null population differentiation was tested by 1000 permutations of the raw data.

### Recombinant analysis

Possible recombination events in the CP gene regions of 133 PRSV isolates from Guangdong (69 isolates) and Hainan (64 isolates) were detected using RDP(R), GENECONV(G), BOOTSCAN(B), MaxChi(M), CHIMAERA(C), SiSCAN(S) and 3SEQ(3) of RDP package software. Results showed that 36 of the isolate sequences (27.07% of total 133 isolates) were identified with a high level of confidence as recombinants (Table 3), which could be divided into two recombination types according to the breakpoints of the sequence alignment. Twenty-eight isolate sequences from Guangdong and four sequences from Hainan (breakpoints at 829–411 bp) were identified as recombinants by all seven methods, which were assigned as type I; four isolate sequences from Guangdong (breakpoints at 854–423 bp) were identified as recombinant by six methods except GENECONV(G), which were assigned as type II. These results showed that the recombinants of the Guangdong isolates (46.3% of 69 isolates) were significantly higher than those of the Hainan isolates (6.3% of 64 isolates), and most of the recombinants belonged to recombination type I.

**Table 3:** List of the isolates of *Papaya ringspot virus* from Guangdong and Hainan with recombinant backgrounds detected by RDP Suites based on the *CP* gene nucleotide sequences.

Recombinant^a^	Break point^b^	Methods^c^	P-value range
**Z5,FM26,GZ30,GZ35,NK11,K6,K9,NO13,T5,T6,TF3,TF4,WS1,Y10,Y9,YJ4,ZC1,ZC2,HZ1,H22,LD1,MZ1,QY1,ZS2,ZS5,ZS6**,SD24,SD26,SD7,SD8	829–411	RGBMCS3	5.33 × 10^−19^ ~1.30 × 10^−5^
**GM1,GM2,GM3,GM4**	854–423	RBMCS3	2.63 × 10^−4^ ~1.68 × 10^−2^

^a^The bold font is stand for Guangdong isolates, while the normal font is stand for Hainan isolates; ^b^the break points listed refer to their position in the alignment; ^c^RDP(R), GENECONV(G), BOOTSCAN(B), MaxChi(M), CHIMAERA(C), SiSCAN(S), 3SEQ(3).

### Phylogenetic analysis

To rule out the interference of recombination in phylogenetic tree construction^25^, we removed the sequences of 36 recombinants and used the remaining 97 CP sequences of the Guangdong and Hainan isolates to construct a phylogenetic tree.

**Figure 4:**
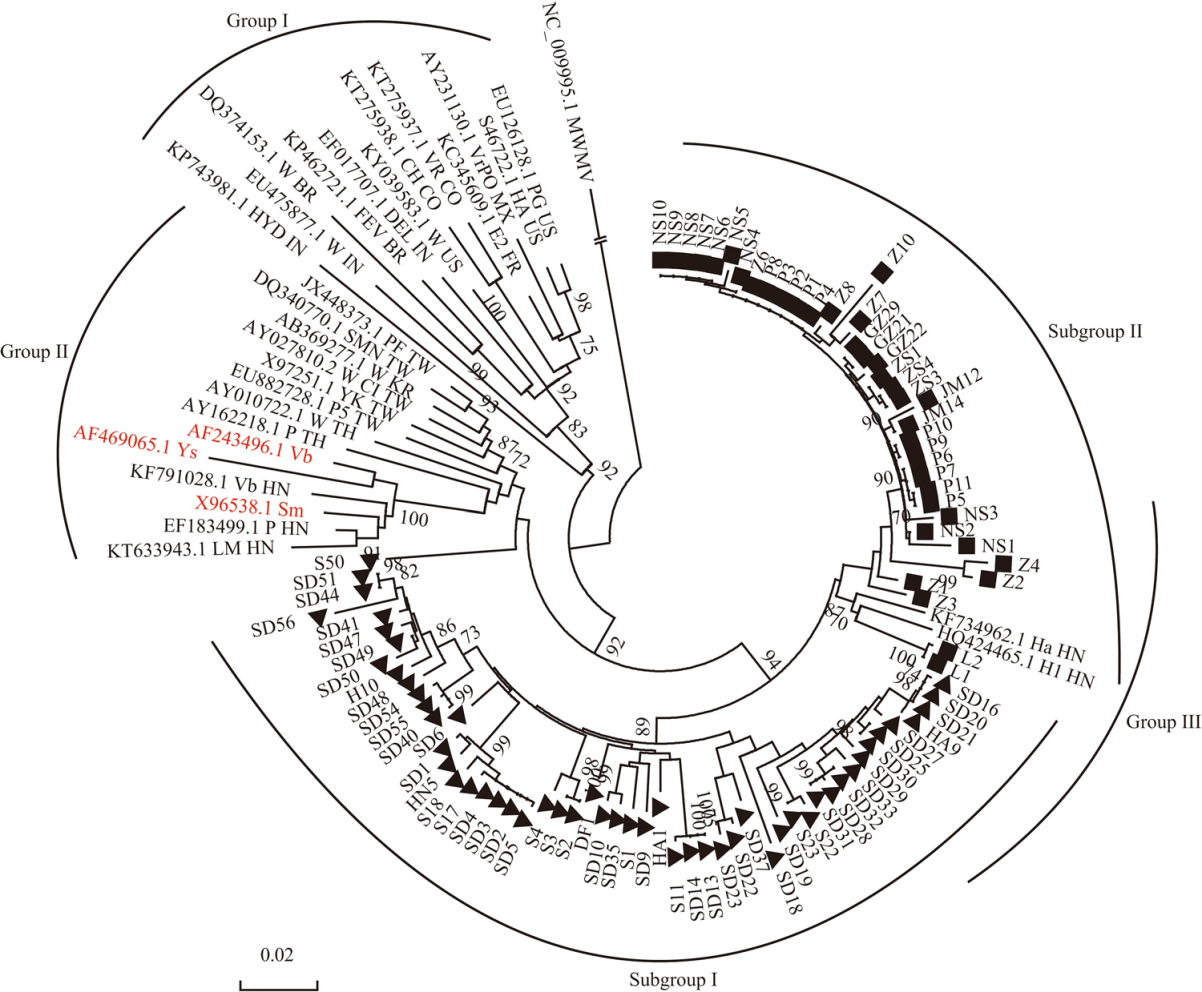
Phylogenetic tree of 97 isolates from Guangdong and Hainan, and 23 isolates derived from GenBank, of *Papaya ringspot virus* reconstructed by Neighbour-Joining method implemented in MEGA 6 based on the nucleotide sequences of *CP* genes. The percentage of replicate trees in which the associated taxa clustered together in the bootstrap test (1000 replicates) is shown next to the branches. The sequenced PRSV as reference genes presented with Genbank accession number followed the region code: HN for Hainan, TH for Thailand, TW for Taiwan, KR for Korean, IN for India, BZ for Brazil, CO for Colombia, FR for France, MX for Mexico and US for USA. ▴ is for Hainan and ▪ is for Guangdong.

The phylogenetic tree results showed that 120 isolates were divided into three groups based on the nucleotide sequences of the CP genes (Fig. 4). All American and Indian isolates were clustered into Group I, whereas Asian isolates from Thailand, South Korea, China including Taiwan and South China were clustered into Group II, including three Hainan isolates (Vb_HN, P_HN and LM_HN) and three previously reported dominant South China strains (Ys, Vb and Sm). All 91 isolates collected from Guangdong and Hainan were clustered into Group III, and Group III was further divided into two subgroups. Most Hainan isolates were grouped into subgroup I, and all Guangdong isolates were grouped into subgroup II.

The genetic distance values between groups and within groups of the isolates are shown in Table 2S. The genetic distance values within three groups ranged from 0.059 ± 0.005 to 0.089 ± 0.005, which were lower than those between groups (0.079 ± 0.006 to 0.115 ± 0.008). In particular, the genetic distance values between Groups III and II and between Groups III and I were 0.115 ± 0.008 and 0.115 ± 0.009, respectively, which were higher than those between Groups II and I (0.079 ± 0.006). This result indicates that Group III is a new emergent lineage of PRSV.

### Selection pressures acting on PRSV CP genes

A large number of selection pressure sites (Table 4) were detected in the 133 CP gene sequences of PRSV from the Guangdong and Hainan isolates with the fixed effects likelihood (FEL), internal branches fixed-effects likelihood (IFEL) and mixed effects model of evolution (MEME) methods in HyPhy package. A total of 92 purifying (negative) (94.8%) and 5 positive selection sites (5.2%) were detected by FEL, and 64 purifying (92.8%) and 5 positive selection pressure sites (7.2%) were detected by IFEL. The number of purifying selection pressure sites was much higher than that of positive selection pressure sites, indicating that the CP genes of PRSV were mainly acted under the pressure of purifying (negative) selection.

**Table 4:** The number of selection pressure sites detected in the 133 *CP* gene sequences of *Papaya ringspot virus* from the Guangdong and Hainan isolates.

Sites*	Purifying		Positive		
	FEL	IFEL	FEL	IFEL	MEME
1611	336	323	1	1	10

^*^Sites, Total number of sites (excluding sites with gaps/missing data). FEL, IFEL, and MEME were estimated using in the HyPhy package.

### Neutrality tests

The Tajima’s D, Fu & Li’s D and Fu & Li’s F values for PRSV Group III from the Guangdong and Hainan isolates were negative, but the data were not significant (Table 5). This result indicates that the PRSV populations in Guangdong and Hainan were relatively stable.

**Table 5:** Neutrality tests of 133 CP gene sequences of *Papaya rinngspot* virus isolates from Guangdong and Hainan.

Region	Tajima’s D	Fu and Li’s D	Fu and Li’s F
GD	−0.181 ns	−0.516 ns	−0.451 ns
HN	−1.318 ns	−0.944 ns	−1.320 ns

ns, not significant; *0.01 < P < 0.05; **0.001 < P < 0.01; ***P < 0.001.

Tajima’s D, Fu & Li’s D and Fu & Li’s F were implemented in Arlequin 3.5.

## Discussion

Papaya is an important fruit crop which is widely cultivated in the tropical and subtropical regions of South China and other countries worldwide. PRSV is the most widespread and damaging virus that infects papaya. The large-scale cultivation and commercialisation of antiviral transgenic papaya have been successful in Hawaii and South China for a long time^17,20,26,27^. However, recent studies have found that PRSV infects ‘Huanong No.1’ transgenic papaya plants in some regions of South China^24^, and the occurrence tendency increases gradually with time. To explain the loss of transgenic papaya resistance against PRSV in South China, we investigated the virus occurrence in ‘Huanong No.1’ papaya plantations in Guangdong and Hainan during 2012–2016, and found that transgenic papaya plants were respectively infected by two kinds of the viruses, PRSV and *Papaya leaf-distortion mosaic virus* (data not shown). This result implied that the genetic structure and population of PRSV in South China may have changed. Zhao *et al*.^23^ analysed 76 PRSV isolates collected from diseased papaya plants of all different papaya cultivars excluding ‘Huanong No.1’ in Hainan in 2010 and inoculated some isolates onto genetically modified papaya seedlings from Hawaii, Taiwan and Guangdong. Results showed that all of these isolates could cause classical symptoms on the transgenic plants at 15 days post-inoculation. In the present study, we inoculated 20 representative isolates from Guangdong and Hainan to transgenic ‘Huanong No.1’ seedlings and observed obvious symptoms at 15 days post-inoculation. These results indicate that the PRSV population in South China changed along with time and with the increased number of cultivated transgenic papaya.

According to the symptom characteristics of PRSV on *C*. *pepo*, Cai *et al*.^18^ classified the virus in four provinces of South China as four stains: Ys with yellow spots on leaves, Vb with gravy bands along veins, Sm with severe mosaic and Lc with leaf curl. In the present study, 20 representative isolates from Guangdong and Hainan were inoculated onto *C*. *pepo*, and the leaves exhibited irregular chlorosis along the leaf veins (Fig. 2). This symptom was obviously different from those caused by the four strains mentioned above. Furthermore, the CP sequence alignment between these strains (Ys, Vb and Sm) and the 133 isolates from Guangdong and Hainan showed that the identities of nucleotides were 86.6%–89.2%, 89.2%–92.4% and 89.2%–92.1%, respectively. This result implied that these 133 isolates were significantly different from Ys in South China but relatively similar to Vb and Sm. The amino acid sequences of 10 representative Guangdong and Hainan isolates were also compared with Ys, Vb and Sm (Fig. 3), and results showed several domain sites that had been changed. In the 10 Guangdong and Hainan isolates, three to four E or K were added in the sites of EK repeats as compared with Ys, Vb and Sm. Bateson *et al*.^28^ and Jain *et al*.^29^ analysed the CP-coding region of PRSV isolates from Vietnam and India, respectively, and found that the number of amino acids also changed in the ‘EK’ region. The EK region is an important component located on the outer surface of the CP^30^ protein and is closely related to the aphid transmission element^31–33^. Mulot *et al*.^34^ revealed that the membrane-bound Ephrin receptor (Eph) in *Myzus persicae* is a novel aphid protein which is involved in the transmission of the *Turnip yellows virus* (TuYV) and further confirmed that the minor capsid protein of TuYV, essential for aphid transmission, was able to bind the external domain of Eph in yeast. Therefore, the addition or deletion of amino acids in the ‘EK’ region may influence aphids on the transmission of PRSV, so that the original dominant strain cannot be effectively and continuously transmitted. After the virus evolves a new ‘EK’ region suitable for aphid transmission, the new isolates gradually disseminate and substitute for the original strains. In addition, the conserved DAG, WCIEN and QMKAAA domains in the CP region were also postulated to be associated with virus transmission by aphids^30,31,33^. Meanwhile, outside the ‘EK’ domain, we found that two amino acid residues (Thr_34_ to Ala_38_, Thr_82_ to Ser_86_) of the 10 representative Guangdong and Hainan isolates were different from the Ys, Vb and Sm strains, 12 amino acids residues (11 at the C-terminal and 1 at the N-terminal) were different from the Ys strain and two sites (both at the N- terminal) were different between the Guangdong and Hainan isolates. The core region of CP is associated with viral assembly^35,36^, plasmodesmatal gating^37^ and cell-to-cell movement^38^. The N- and C-terminals of CP are related to the long-distance movement and virus systemic movement. A point mutation (Ser_47_ to Pro, Asp_5_ to Lys) in the N-terminal of CP alters the ability of *Pea seed-borne mosaic virus* or *Tobacco vein mottling virus* to infect *Chenopodium quinoa*^39^ or tobacco^40^, respectively. A single substitution (Ser_7_ to Gly) at the CP N-terminus reduces virus accumulation at 10-fold but restores aphid transmissibility of *Potato virus* A^41^. In the present study, most of the variation sites were located at the N- or C-terminals of CP, indicating that their changes may lead to the differentiation of the concentrations or systemic movement capacity of PRSV isolates in papaya. In addition, Zamora *et al*.^42^ analysed the RNA binding mode in *Potyvirus* and revealed that mutations in the conserved Arg and Asp residues of the CP impaired *in vitro* assembly of the *potyvirus* and blocked the assembly and cell-to-cell movement of the *potexvirus* in plants. In present study, we speculated that the CP of PRSV might have RNA binding function. Thus, various patterns of conserved elements and the recombinations may affect the RNA binding function. Further studies should be conducted for verification.

Phylogenetic analysis of PRSV CP nucleotide sequences from South China and other countries were performed. Results showed that PRSV isolates were distinctly divided into three groups (Fig. 4). Almost all Guangdong and Hainan isolates were clustered into Group III, although they belonged to two subgroups based on geographical locations. However, Ys, Vb and Sm in South China were clustered into Group II, along with Asian isolates from Thailand, South Korea and China Taiwan. The genetic distance values between Groups III and II and between Groups III and I were 0.115 ± 0.008 and 0.115 ± 0.009, respectively, which were higher than those between Groups II and I (0.079 ± 0.006), indicating that Group III belongs to a new lineage. We suppose that the long-term cultivation of genetically modified papaya in Guangdong and Hainan may have led to the emergence of the new PRSV isolate lineage.

Recombination is an important factor to promote virus evolution, which can increase genomic biodiversity^43^, reduce mutations in specific genome sequences^44^ and restore genome integrity^45^. In addition, recombination enhances the virulence of the virus and extends its host ranges^46^. This process has been found in many *Potyvirus* species^47–50^. The recombination hotspot of PRSV is the *P1* gene, followed by P3 and CI, HC-Pro and CP^51^. According to the two breakpoints of nucleotides (746–853 and 591–861), Mangrauthia *et al*.^51^ discovered two recombination types in the CP region. In present study, 36 (27.1%) of the 133 PRSV Guangdong and Hainan isolates showed clear recombination in the CP region. Two recombination types were also detected, but the recombination sites were respectively located in positions 829–411 and 854–423, which were different from those of the types above. The breakpoints found in the former^51^ are mainly located at the C-terminal of CP, whereas those in the present study are located at the N- and C-terminals of CP, respectively. We also compared the amino acid sequences of CP from the Guangdong and Hainan isolates and found that the variation sites were mostly located in the N- and C-terminals of CP. Thus, we supposed that the virus can reduce harmful mutations and maintain genome stability by variation or recombination of the CP region, which probably led to the rapid spread of new group populations.

In the present study, the CP genes of the PRSV Guangdong and Hainan isolates exhibited a high degree of genetic diversity. Three indices were used to test the population differentiation between the Guangdong and Hainan isolates based on the PRSV CP nucleotide sequences. *K*_*ST*_ and *F*_*ST*_ may measure the relative proportions of total genetic diversity attributable to among populations, and range from 0.00 to 1.00. A value of 1.00 for *K*_*ST*_ or *F*_*ST*_ indicates that populations are completely differentiated, while a value of 0.00 indicates the populations are identical^52^. *K*_*ST*_ or *F*_*ST*_ values between 0.15 or 0.25 indicate high population differentiation, and values greater than 0.25 indicate very high genetic differentiation among populations^53^. The *Snn* value may indicate the frequency of the most similar sequences in the same population, and *Snn* values close to 1.0 indicate that the population is highly differentiated while values near 0.5 ^54^ indicate that the population is identical. In the current study, the *K*_*ST*_ and *F*_*ST*_ values were close to or higher than 0.15 and the *Snn* values were close to 1, suggesting that the Guangdong and Hainan isolates in this study composed a highly differentiated new population of PRSV in South China.

Positive selection of virus population may endow the virus more fitness to adapt a new hosts or environments, whereas rapid divergence driven by positive selection has been seldom demonstrated^55^. Similar to other viral genes, majority of the codons in the CP genes of the Guangdong and Hainan isolates in the present study were detected to be under the status of negative (purifying) selection. This result suggests that most of the codon mutations in the PRSV genome are detrimental and thus are easily eliminated by natural selection. In this case, the selection may come from living environment differences between the two regions, such as differences in papaya cultivars and climatic conditions. After a long-term tracking survey of transgenic cultivars in Guangdong and Hainan, we found that ‘Huanong No.1’ was the dominant cultivar grown in Guangdong, while more various cultivars from other countries and regions, including ‘Huanong No.1’, were grown in Hainan. Guangdong Province is located at 20° 13′N–25° 31′N, 109° 39′E–117° 19′E, while Hainan Island is located at 19° 20′N–20° 10′N, 108° 21′E–111° 03′E. The former belongs to subtropical and tropical monsoon climate with an annual average temperature of 19–23 °C, whereas the latter belongs to tropical monsoon climate with an annual average temperature of 23–25 °C. These two regions were separated by Qiongzhou Strait, resulting in a certain geographical isolation^56^. These differences may lead to the gradual differentiation of PRSV between the Guangdong and Hainan isolates and eventually induce those isolates to evolve into two subgroups.

Tajima^57^ has developed a statistical method for testing the neutral mutation hypothesis by using the average number of nucleotide differences and the number of segregating sites. If a population experiences a bottleneck or balance, Tajima’s D values are significantly higher than 0; if a population experiences a size expansion or directional selection, Tajima’s D values are significantly less than 0. Since both balance and directional selection fall into the category of positive selection, natural selection may be accepted as long as the Tajima’s D value deviates significantly from 0, whereas the null hypothesis that neutral selection cannot be rejected when the Tajima’s D value does not significantly deviate from 0. Fu and Li^58^ proposed Fu & Li’s D and Fu & Li’s F test for neutral selection in comparison with Tajima’s D test. The proposed method considers the availability of external branches so that a rooted tree can be constructed for a given set of DNA sequences. The number of external mutations is different from its neutral expected values in the presence of selection, whereas the number of internal mutations is only slightly affected by the presence of selection. In the current study, the results of neutrality tests of Tajima’s D, Fu & Li’s D and Fu & Li’s F of the isolates from South China showed that they were all negative. No significant difference was found between the Guangdong and Hainan isolates, indicating that these two population groups did not significantly deviate from the neutral evolution and may have evolved into two relatively stable populations.

In summary, we analysed and confirmed the population characteristics of PRSV isolates in South China by collecting transgenic ‘Huanong No.1’ papaya samples from Guangdong and Hainan during 2012–2016. These isolates were highly differentiated from the previously reported strains of South China and therefore have led to the formation of a new emergent lineage which can infect genetically engineered papaya grown widely in South China.

## Methods

### Samples and locations

Fresh leaf samples were collected from susceptible transgenic ‘Huanong No.1’ papaya from 10 counties in Guangdong (Guangzhou, Jiangmen, Zhanjiang, Zhongshan, Foshan, Shenzhen, Qingyuan, Huizhou, Luoding and Meizhou) and six counties in Hainan (Sanya, Ledong, Dongfang, Changjiang, Haikou and Lingshui) from 2012 to 2016 (Fig. 1, Table 1S). More than 10 diseased samples were randomly collected from papaya farms to investigate the virus occurrence. Samples were stored on ice-cold package, taken back to the laboratory, frozen in liquid nitrogen and then stored at −80 °C.

### Artificial inoculation

The samples were divided into four groups according to geographical regions (Guangdong and Hainan) and symptomatic characteristics (mosaic and distortion), and 0.1 M phosphate buffer (pH 7.0) was used to grind leaf samples as inoculum. Five samples were selected from each group, inoculated firstly into *C*. *quinoa* for purification and then inoculated into squash cultivar ‘Zaoqingyidai’ (*C*. *pepo*) and transgenic papaya cultivar ‘Huanong No.1’^18^. Phosphate buffer was administered in the control group. Symptoms were recorded at 7–15 days post-inoculation from 15 plants per treatment, and the experiment was repeated thrice. The plants were maintained in a greenhouse at 25 °C under natural light. The RNAs were extracted from susceptible squash and papaya leaves, and the coat protein genes of PRSV were cloned to compare with the sequences of the original inoculated PRSV isolates. Results of this experiment were used to verify Koch’s Postulate and compare the isolates’ biological characteristics.

### Viral RNA extraction, cDNA cloning and sequencing

Total RNAs were extracted from the papaya leaves with Tiangen Trizol Kit in accordance with the instructions of the manufacturer (TianGen, Beijing, China). Primers (CP-F: ATGTCCAAGAATGAAGCT, CP-R:TTAGTTGCGCATACCCA) were designed based on the conserved region sequences of four PRSV isolates (HQ424465.1, X9725.1, KF734962.1 and EF183499.1) registered in GenBank using Primer Premier v6.0 software (PREMIER Biosoft International, Canada). The RT-PCR reaction mixture contained 1 μL of total RNAs, 1.25 μL of each primer (10 μΜ), 25 μL of 2x One Step MasterMix, 2 μL of 25x Enzyme Mix and nuclease-free water to a final volume of 50 µL (TianGen, Beijing, China). The reaction program was performed under following conditions: 45 °C for 30 min; 95 °C for 3 min; 35 cycles of 94 °C for 30 sec, 55 °C for 1 min and 72 °C for 30 sec; and a final extension for 5 min at 72 °C. PCR products were separated by 1% agarose gel electrophoresis, extracted and then purified from the gel with a Tiangen gel extraction kit (TianGen, Beijing, China). The purified products were cloned into the pMD18-T vector (Takara) in accordance with the manufacturer’s protocol and then transformed into *Escherichia coli* DH5α competent cells. Three positive clones from each transformation were selected and sequenced (Abi 3130xl Genetic Analyzer; Hitachi). All sequences generated in this study were deposited in the GenBank database. In addition, 23 nucleotide sequences of different isolates from different countries and regions were downloaded from GenBank as the reference sequences (Table 1S).

### Genetic diversity and population genetic differentiation

Nucleotide sequence identity matrices were calculated using BioEdit software^59^ after all gaps were removed. Boxplots of the 133 Guangdong and Hainan isolates and previously reported dominant strains (Ys, Vb and Sm) in South China were mapped using R 2.9.1 (R Project for Statistical Computing website). Haplotype and nucleotide diversities were estimated using DnaSP 5.0^60^. Haplotype diversity refers to the frequency and number of haplotypes in the population. Nucleotide diversity estimates the average pairwise differences among the sequences. Genetic differentiation among populations was calculated by *F*_*ST*_ using Arlequin 3.5^61^. Genetic differentiation among populations was also calculated by *K*_*ST*_ and *S*_*nn*_ using DnaSP 5.10 ^52,54,60^. The hypothesis of deviation from the null population differentiation was tested by 1000 permutations of the raw data.

### Recombination analysis

The nucleotide sequences of 133 PRSV isolates from Guangdong and Hainan were subjected to recombination analysis using seven methods (RDP, GENECONV, BOOTSCAN, MaxChi, CHIMAERA, SiSCAN and 3SEQ) implemented in RDP v4.71 software^62^. The probability of a putative recombination event was corrected by a Bonferroni procedure with a cutoff of p-value less than 0.01. To avoid misidentification, only events supported by at least four of the seven methods were considered to be recombinants. Recombinants were excluded from the reconstruction of phylogenetic trees.

### Phylogenetic analysis

The nucleotide sequences of the CP genes were aligned using the Muscle algorithm^63^ implemented in MEGA 6^64^. Phylogenetic tree of PRSV isolates excluding the recombinants was reconstructed by the Neighbour–Joining method^65^ implemented in MEGA 6. The CP gene of one isolate of *Moroccan watermelon mosaic virus* (MWMV) (Accession No. NC_009995.1) was used as a outgroup^8^. Bootstrap analysis was repeated 1000 times to evaluate the significance of the internal branches. The intra- and inter-group genetic distances were calculated using MEGA 6.

### Selection pressure analysis

The selection pressure was estimated by d_N_/d_S_ ratio, where d_N_ represents the average number of non-synonymous substitutions per non-synonymous site and d_S_ represents the average number of synonymous substitutions per synonymous site. HyPhy 2.10b^66^ was used to identify the nucleotide sites in CP cistrons that may be involved in PRSV adaptation. Three codon-based approaches, FEL, IFEL and MEME, were included in the HyPhy package^67–69^.

### Neutrality test

Tajima’s D, Fu & Li’s D and Fu & Li’s F of Arlequin 3.5^61^ were used to conduct neutrality test on the PRSV isolates from Guangdong and Hainan to clarify the historical dynamics of both populations. Tajima’s D, Fu & Li’s D and Fu & Li’s values assume that all mutations are selectively neutral. Tajima’s D test identifies evolutionary events such as population expansion, bottleneck effects and natural selection by comparing the numbers of segregating sites and the average number of nucleotide differences^57^. Fu & Li’s D and Fu & Li’s F values are sensitive to population expansion and are usually negative when the population is expanded^70^.

## Electronic supplementary material

Supplementary Information
