# Supplementary material for: Characterization of Papaya ringspot virus isolates infecting transgenic papaya ‘Huanong No.1’ in South China
Source: Sci Rep. 2018 May 29;8:8206. doi: 10.1038/s41598-018-26596-x (PMC5974079; doi:10.1038/s41598-018-26596-x)
Supplement: Supplementary file 1 — Supplementary Information [file 41598_2018_26596_MOESM1_ESM.doc]

**Characterization of *Papaya ringspot virus* isolates infecting transgenic papaya ‘Huanong No.1’ in South China**

Zilin Wu, Cuiping Mo, Shuguang Zhang, Huaping Li*

State Key Laboratory of Conservation and Utilization of Subtropical Agro-bioresources, Guangdong Province Key Laboratory of Microbial Signals and Disease Control, College of Agriculture, South China Agricultural University, Guangzhou 510642, China.

*Correspondent author:

E-mail: [huaping@scau.edu.cn](mailto:huaping@scau.edu.cn)


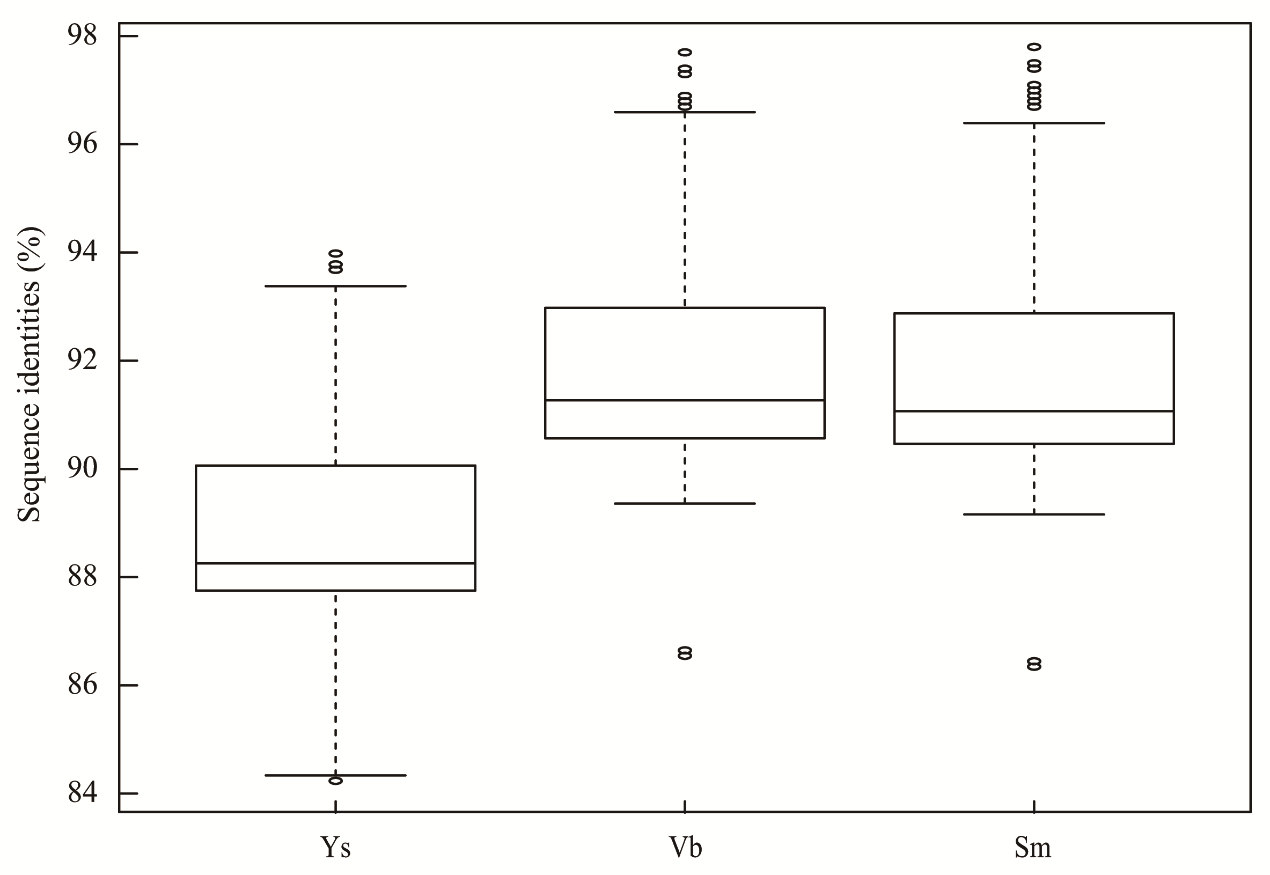


**Supplementary Fig.1S Boxplot graphs show nucleotide sequence identity scores of the coat protein genes of *Papaya ringspot virus* between three previously reported dominant strains in South China (Ys, Vb, and Sm) and 133 Guangdong and Hainan isolates.** The graphs was mapped using R 2.9.1(R Project for Statistical Computing website).

| **Supplementary Table 1S PRSV isolates in this study** | | | | | |
| --- | --- | --- | --- | --- | --- |
| **Isolates** | **Country** | **Host** | **Date** | **Accession Number** | **Reference** |
| SD1 | China:Hainan,Sanya | *Carica papaya* | 2012 | MG564572 | This study |
| SD2 | China:Hainan,Sanya | *Carica papaya* | 2012 | MG564575 | This study |
| SD3 | China:Hainan,Sanya | *Carica papaya* | 2012 | MG564574 | This study |
| SD4 | China:Hainan,Sanya | *Carica papaya* | 2012 | MG564573 | This study |
| SD5 | China:Hainan,Sanya | *Carica papaya* | 2012 | MG564576 | This study |
| SD6 | China:Hainan,Sanya | *Carica papaya* | 2012 | MG564579 | This study |
| SD7 | China:Hainan,Sanya | *Carica papaya* | 2012 | MG564624 | This study |
| SD8 | China:Hainan,Sanya | *Carica papaya* | 2012 | MG564623 | This study |
| SD9 | China:Hainan,Sanya | *Carica papaya* | 2012 | MG564595 | This study |
| SD10 | China:Hainan,Sanya | *Carica papaya* | 2012 | MG564583 | This study |
| SD13 | China:Hainan,Sanya | *Carica papaya* | 2012 | MG564598 | This study |
| SD14 | China:Hainan,Sanya | *Carica papaya* | 2012 | MG564597 | This study |
| SD16 | China:Hainan,Sanya | *Carica papaya* | 2012 | MG564612 | This study |
| SD18 | China:Hainan,Sanya | *Carica papaya* | 2012 | MG564617 | This study |
| SD19 | China:Hainan,Sanya | *Carica papaya* | 2012 | MG564611 | This study |
| SD20 | China:Hainan,Sanya | *Carica papaya* | 2012 | MG564609 | This study |
| SD21 | China:Hainan,Sanya | *Carica papaya* | 2012 | MG564608 | This study |
| SD22 | China:Hainan,Sanya | *Carica papaya* | 2012 | MG564615 | This study |
| SD23 | China:Hainan,Sanya | *Carica papaya* | 2012 | MG564614 | This study |
| SD24 | China:Hainan,Sanya | *Carica papaya* | 2012 | MG564621 | This study |
| SD25 | China:Hainan,Sanya | *Carica papaya* | 2012 | MG564594 | This study |
| SD26 | China:Hainan,Sanya | *Carica papaya* | 2012 | MG564622 | This study |
| SD27 | China:Hainan,Sanya | *Carica papaya* | 2012 | MG564596 | This study |
| SD28 | China:Hainan,Sanya | *Carica papaya* | 2012 | MG564604 | This study |
| SD29 | China:Hainan,Sanya | *Carica papaya* | 2012 | MG564590 | This study |
| SD30 | China:Hainan,Sanya | *Carica papaya* | 2012 | MG564593 | This study |
| SD31 | China:Hainan,Sanya | *Carica papaya* | 2012 | MG564600 | This study |
| SD32 | China:Hainan,Sanya | *Carica papaya* | 2012 | MG564599 | This study |
| SD33 | China:Hainan,Sanya | *Carica papaya* | 2012 | MG564610 | This study |
| S1 | China:Hainan,Sanya | *Carica papaya* | 2014 | MG564589 | This study |
| S2 | China:Hainan,Sanya | *Carica papaya* | 2014 | MG564586 | This study |
| S3 | China:Hainan,Sanya | *Carica papaya* | 2014 | MG564585 | This study |
| S4 | China:Hainan,Sanya | *Carica papaya* | 2014 | MG564591 | This study |
| S11 | China:Hainan,Sanya | *Carica papaya* | 2014 | MG564603 | This study |
| S17 | China:Hainan,Sanya | *Carica papaya* | 2014 | MG564577 | This study |
| S22 | China:Hainan,Sanya | *Carica papaya* | 2014 | MG564628 | This study |
| S23 | China:Hainan,Sanya | *Carica papaya* | 2014 | MG564605 | This study |
| S50 | China:Hainan,Sanya | *Cucurbita moschata* | 2014 | MG564629 | This study |
| HA1 | China:Hainan,Sanya | *Carica papaya* | 2016 | MG564584 | This study |
| HA9 | China:Hainan,Sanya | *Carica papaya* | 2016 | MG564606 | This study |
| SD35 | China:Hainan,Ledong | *Carica papaya* | 2012 | MG564587 | This study |
| SD37 | China:Hainan,Ledong | *Carica papaya* | 2012 | MG564607 | This study |
| SD56 | China:Hainan,Ledong | *Carica papaya* | 2012 | MG564618 | This study |
| S18 | China:Hainan,Ledong | *Carica papaya* | 2014 | MG564578 | This study |
| SD40 | China:Hainan,Dongfang | *Carica papaya* | 2012 | MG564582 | This study |
| SD41 | China:Hainan,Dongfang | *Carica papaya* | 2012 | MG564592 | This study |
| SD44 | China:Hainan,Dongfang | *Carica papaya* | 2012 | MG564602 | This study |
| SD46 | China:Hainan,Dongfang | *Carica papaya* | 2012 | MG564620 | This study |
| SD47 | China:Hainan,Dongfang | *Carica papaya* | 2012 | MG564613 | This study |
| SD48 | China:Hainan,Dongfang | *Carica papaya* | 2012 | MG564616 | This study |
| SD49 | China:Hainan,Dongfang | *Carica papaya* | 2012 | MG564588 | This study |
| SD50 | China:Hainan,Dongfang | *Carica papaya* | 2012 | KT895257 | This study |
| SD51 | China:Hainan,Dongfang | *Carica papaya* | 2012 | MG564601 | This study |
| SD52 | China:Hainan,Dongfang | *Carica papaya* | 2012 | MG564619 | This study |
| SD54 | China:Hainan,Dongfang | *Carica papaya* | 2012 | MG564581 | This study |
| SD55 | China:Hainan,Dongfang | *Carica papaya* | 2012 | MG564580 | This study |
| HN5 | China:Hainan,Dongfang | *Carica papaya* | 2012 | MG564627 | This study |
| H10 | China:Hainan,Dongfang | *Carica papaya* | 2012 | MG564626 | This study |
| DF1 | China:Hainan,Dongfang | *Carica papaya* | 2014 | MG564625 | This study |
| H22 | China:Guangdong,Jiangmen | *Carica papaya* | 2012 | MG564566 | This study |
| JM12 | China:Guangdong,Jiangmen | *Carica papaya* | 2013 | MG564570 | This study |
| JM14 | China:Guangdong,Jiangmen | *Carica papaya* | 2013 | MG564569 | This study |
| NO13 | China:Guangdong,Guangzhou | *Carica papaya* | 2014 | MG564511 | This study |
| WS14 | China:Guangdong,Guangzhou | *Carica papaya* | 2014 | MG564567 | This study |
| NC14 | China:Guangdong,Guangzhou | *Carica papaya* | 2014 | MG564568 | This study |
| NS1 | China:Guangdong,Guangzhou | *Carica papaya* | 2015 | MG564512 | This study |
| NS2 | China:Guangdong,Guangzhou | *Carica papaya* | 2015 | MG564513 | This study |
| NS3 | China:Guangdong,Guangzhou | *Carica papaya* | 2015 | MG564514 | This study |
| NS4 | China:Guangdong,Guangzhou | *Carica papaya* | 2015 | MG564515 | This study |
| NS5 | China:Guangdong,Guangzhou | *Carica papaya* | 2015 | MG564516 | This study |
| NS6 | China:Guangdong,Guangzhou | *Carica papaya* | 2015 | MG564517 | This study |
| NS7 | China:Guangdong,Guangzhou | *Carica papaya* | 2015 | MG564518 | This study |
| NS8 | China:Guangdong,Guangzhou | *Carica papaya* | 2015 | MG564519 | This study |
| NS9 | China:Guangdong,Guangzhou | *Carica papaya* | 2015 | MG564520 | This study |
| NS10 | China:Guangdong,Guangzhou | *Carica papaya* | 2015 | MG564521 | This study |
| P1 | China:Guangdong,Guangzhou | *Carica papaya* | 2015 | MG564522 | This study |
| P2 | China:Guangdong,Guangzhou | *Carica papaya* | 2015 | MG564523 | This study |
| P3 | China:Guangdong,Guangzhou | *Carica papaya* | 2015 | MG564524 | This study |
| P4 | China:Guangdong,Guangzhou | *Carica papaya* | 2015 | MG564525 | This study |
| P5 | China:Guangdong,Guangzhou | *Carica papaya* | 2015 | MG564526 | This study |
| P6 | China:Guangdong,Guangzhou | *Carica papaya* | 2015 | MG564527 | This study |
| P7 | China:Guangdong,Guangzhou | *Carica papaya* | 2015 | MG564528 | This study |
| P8 | China:Guangdong,Guangzhou | *Carica papaya* | 2015 | MG564529 | This study |
| P9 | China:Guangdong,Guangzhou | *Carica papaya* | 2015 | MG564530 | This study |
| P10 | China:Guangdong,Guangzhou | *Carica papaya* | 2015 | MG564531 | This study |
| P11 | China:Guangdong,Guangzhou | *Carica papaya* | 2015 | MG564532 | This study |
| T5 | China:Guangdong,Guangzhou | *Carica papaya* | 2015 | MG564534 | This study |
| T6 | China:Guangdong,Guangzhou | *Carica papaya* | 2015 | MG564535 | This study |
| TF3 | China:Guangdong,Guangzhou | *Carica papaya* | 2015 | MG564536 | This study |
| TF4 | China:Guangdong,Guangzhou | *Carica papaya* | 2015 | MG564537 | This study |
| Y9 | China:Guangdong,Guangzhou | *Carica papaya* | 2015 | MG564538 | This study |
| Y10 | China:Guangdong,Guangzhou | *Carica papaya* | 2015 | MG564539 | This study |
| ZC1 | China:Guangdong,Guangzhou | *Carica papaya* | 2015 | MG564549 | This study |
| ZC2 | China:Guangdong,Guangzhou | *Carica papaya* | 2015 | MG564550 | This study |
| K6 | China:Guangdong,Guangzhou | *Carica papaya* | 2015 | MG564504 | This study |
| K9 | China:Guangdong,Guangzhou | *Carica papaya* | 2015 | MG564505 | This study |
| K12 | China:Guangdong,Guangzhou | *Carica papaya* | 2015 | MG564506 | This study |
| GZ21 | China:Guangdong,Guangzhou | *Carica papaya* | 2016 | MG564562 | This study |
| GZ22 | China:Guangdong,Guangzhou | *Carica papaya* | 2016 | MG564563 | This study |
| GZ29 | China:Guangdong,Guangzhou | *Carica papaya* | 2016 | MG564564 | This study |
| GZ30 | China:Guangdong,Guangzhou | *Carica papaya* | 2016 | MG564565 | This study |
| GZ35 | China:Guangdong,Guangzhou | *Carica papaya* | 2016 | MG564566 | This study |
| FM26 | China:Guangdong,Guangzhou | *Carica papaya* | 2016 | MG564557 | This study |
| Z1 | China:Guangdong,Zhanjiang | *Carica papaya* | 2015 | MG564540 | This study |
| Z2 | China:Guangdong,Zhanjiang | *Carica papaya* | 2015 | MG564541 | This study |
| Z3 | China:Guangdong,Zhanjiang | *Carica papaya* | 2015 | MG564542 | This study |
| Z4 | China:Guangdong,Zhanjiang | *Carica papaya* | 2015 | MG564543 | This study |
| Z5 | China:Guangdong,Zhanjiang | *Carica papaya* | 2015 | MG564544 | This study |
| Z6 | China:Guangdong,Zhanjiang | *Carica papaya* | 2015 | MG564545 | This study |
| Z7 | China:Guangdong,Zhanjiang | *Carica papaya* | 2015 | MG564546 | This study |
| Z8 | China:Guangdong,Zhanjiang | *Carica papaya* | 2015 | MG564547 | This study |
| L1 | China:Guangdong,Zhanjiang | *Carica papaya* | 2016 | MG564507 | This study |
| L2 | China:Guangdong,Zhanjiang | *Carica papaya* | 2016 | MG564508 | This study |
| ZS1 | China:Guangdong,Zhongshan | *Carica papaya* | 2015 | MG564540 | This study |
| ZS2 | China:Guangdong,Zhongshan | *Carica papaya* | 2015 | MG564541 | This study |
| ZS3 | China:Guangdong,Zhongshan | *Carica papaya* | 2015 | MG564542 | This study |
| ZS4 | China:Guangdong,Zhongshan | *Carica papaya* | 2015 | MG564543 | This study |
| ZS5 | China:Guangdong,Zhongshan | *Carica papaya* | 2015 | MG564544 | This study |
| ZS6 | China:Guangdong,Zhongshan | *Carica papaya* | 2015 | MG564545 | This study |
| GM1 | China:Guangdong,Foshan | *Carica papaya* | 2015 | MG564558 | This study |
| GM2 | China:Guangdong,Foshan | *Carica papaya* | 2015 | MG564559 | This study |
| GM3 | China:Guangdong,Foshan | *Carica papaya* | 2015 | MG564560 | This study |
| GM4 | China:Guangdong,Foshan | *Carica papaya* | 2015 | MG564561 | This study |
| Z10 | China:Guangdong,Shenzhen | *Carica papaya* | 2015 | MG564548 | This study |
| QY1 | China:Guangdong,Qingyuan | *Carica papaya* | 2015 | MG564533 | This study |
| HZ1 | China:Guangdong,Huizhou | *Carica papaya* | 2016 | MG564503 | This study |
| LD1 | China:Guangdong,Luoding | *Carica papaya* | 2015 | MG564509 | This study |
| MZ1 | China:Guangdong,Meizhou | *Carica papaya* | 2016 | MG564510 | This study |
| Ys | China | *Carica papaya* | 1991 | AF469065.1 | 1 |
| Sm | China | *Carica papaya* | 1996 | X96538.1 | Unpublished |
| Vb | China | *Carica papaya* | 2000 | AF243496.1 | Unpublished |
| P_HN | China:Hainan,Haikou | *Carica papaya* | 2006 | EF183499.1 | 2 |
| H1_HN | China:Hainan,Haikou | *Carica papaya* | 2010 | HQ424465.1 | 3 |
| Ha_HN | China:Hainan,Haikou | *Carica papaya* | 2011 | KF734962.1 | 4 |
| Vb_HN | China: Hainan,Haikou | *Carica papaya* | 2013 | KF791028.1 | 5 |
| LM_HN | China:Hainan,Lingshui | *Carica papaya* | 2015 | KT633943.1 | Unpublished |
| YK_TW | China:Taiwan | *Carica papaya* | 1996 | X97251.1 | 6 |
| W_CI_TW | China:Taiwan | *Cucumis metuliferus* | 2001 | AY027810.2 | Unpublished |
| SMN_TW | China:Taiwan | *Carica papaya* | 2005 | DQ340770.1 | Unpublished |
| P5_TW | China:Taiwan | *Carica papaya* | 2007 | EU882728.1 | Unpublished |
| PF_TW | China:Taiwan | *Carica papaya* | 2012 | JX448373.1 | Unpublished |
| DEL_IN | India | *Carica papaya* | 2006 | EF017707.1 | 7 |
| W_IN | India | *Cucurbites* | 2008 | EU475877.1 | 8 |
| HYD_IN | India | *Carica papaya* | 2015 | KP743981.1 | Unpublished |
| W_TH | Thailand | *Cucurbites* | 2000 | AY010722.1 | 9 |
| P_TH | Thailand | *Carica papaya* | 2002 | AY162218.1 | Unpublished |
| W_BR | Brazil | *Cucurbita pepo* | 2006 | DQ374153.1 | 10 |
| FEV_BR | Brazil | *Fevillea cordifolia* | 2012 | KP462721.1 | Unpublished |
| W_US | USA | *Lagenaria siceraria* | 2010 | KY039583.1 | 11 |
| HA_US | USA | *Carica papaya* | 1992 | S46722.1 | 12 |
| PG_US | USA | *Carica papaya* | 2007 | EU126128.1 | Unpublished |
| CH_CO | Colombia | *Carica papaya* | 2014 | KT275938.1 | 13 |
| VR_CO | Colombia | *Carica papaya* | 2014 | KT275937.1 | 13 |
| VrPO_MX | Mexico | *Carica papaya* | 2003 | AY231130.1 | 14 |
| E2_FR | France | *Cucurbita pepo* | 1979 | KC345609.1 | 15,16 |
| W_KR | South Korea | *Cucumis sativus* | 2007 | AB369277.1 | Unpublished |

***Supplementary Table 2S Genetic distances between and within variant groups of Papaya ringspot virus CP gene.***

| **Phylogroup** | **Between groups** | | **Within groups** |
| --- | --- | --- | --- |
|  | **Group I** | **Group II** |  |
| Group I |  |  | 0.059±0.005 |
| Group II | 0.079±0.006 |  | 0.065±0.005 |
| Group III | 0.115±0.009 | 0.115±0.008 | 0.089±0.005 |

The intra- and inter-group genetic distances were calculated by MEGA 6.

**Supplementary References:**

**1.** Ye, C. & Ye, Y. Sequencing and construction of plant expression vector of coat protein gene from Ys strain of *Papaya ringspot virus*. *Virologica Sinica*. **1,** 008 (1997).

**2.** Lu, Y. W. *et al*. Complete genomic sequence of a *Papaya ringspot virus* isolate from Hainan Island, China. *Arch Virol*. **153,** 991-993 (2008).

**3.** Shen, W. *et al*. First report of mixed infection of *Papaya ringspot virus* and *Papaya leaf distortion mosaic virus* on Carica papaya L. *J Plant Pathol*. **96,** 121 (2014).

**4.** Zhang, Y. *et al*. Complete genome of Hainan *Papaya ringspot virus* using small RNA deep sequencing. *Virus Genes*. **48,** 502-508 (2014).

**5.** Zhao, G. *et al*. Complete genome sequence of *Papaya ringspot virus* isolated from genetically modified papaya in Hainan Island, China. *Genome Announcements*. **3,** e01056-15 (2015).

**6.** Wang, C. H. & Yeh, S. D. Divergence and conservation of the genomic RNAs of Taiwan and Hawaii strains of *Papaya ringspot potyvirus*. *Arch Virol*. **142,** 271-285 (1997).

**7.** Parameswari, B., Mangrauthia, S. K., Praveen, S. & Jain, R. K. Complete genome sequence of an isolate of *Papaya ringspot virus* from India. *Arch Virol*. **152,** 843-845 (2007).

**8.** Mangrauthia, S., Parameswari, B. R. & Praveen, S. Role of genetic recombination in the molecular architecture of *Papaya ringspot virus*. *Biochem Genet*. **46,** 835-846 (2008).

**9.** Attasart, P., Charoensilp, G., Kertbundit, S., Panyim, S. & Juricek, M. Nucleotide sequence of a Thai isolate of *Papaya ringspot virus* type W. *Acta Virol*. **46,** 241-246 (2002).

**10.** Inouenagata, A. K. *et al*. Genome analysis of a severe and a mild isolate of *Papaya ringspot virus*-type W found in Brazil. *Virus Genes*. **35,** 119-127 (2007).

**11.** Ali, A. First complete genome sequence of *Papaya ringspot virus*-W isolated from a gourd in the United States. *Genome Announcements*. **5,** e01434-16 (2017).

**12.** Yeh, S. D. *et al*. Complete nucleotide sequence and genetic organization of *Papaya ringspot virus* RNA. *J Gen Virol*. **73,** 2531-2541 (1992).

**13.** Ortiz-Rojas, L. Y. & Chaves-Bedoya, G. Molecular characterization of two *Papaya ringspot virus* isolates that cause devastating symptoms in Norte De Santander, Colombia. *Eur J Plant Pathol*. **148,** 883-894 (2017).

**14.** Noa-Carrazana, J. C., González-De-León, D. & Silva-Rosales, L. Molecular characterization of a severe isolate of *Papaya ringspot virus* in Mexico and its relationship with other isolates. *Virus Genes*. **35,** 109-117 (2007).

**15.** Romay, G., Lecoq, H. & Desbiez, C. *Zucchini tigré mosaic virus* is a distinct potyvirus in the *Papaya ringspot virus* cluster: molecular and biological insights. *Arch Virol*. **159,** 277-289 (2014).

**16.** Lecoq, H., Lot, H. & Pitrat, M. First identification of *Watermelon mosaic virus* type 1 (WMV1) in Southeastern France. *Agronomie*. (1982).
